# Delayed presentation of retained renal shrapnel: a case report and literature review

**DOI:** 10.1093/jscr/rjag764

**Published:** 2026-08-31

**Authors:** Rawa Bapir, Ahmed Mohammed Abdalqadir, Ismaeel Aghaways, Bryar Othman Muhammed, Farman M Faraj, Soran H Tahir, Nali H Hama, Zheer T Hamasalih, Berun A Abdalla, Fahmi H Kakamad

**Affiliations:** Department of Urology, Smart Health Tower, Madam Mitterrand Street, Sulaymaniyah 46001, Iraq; Department of Urology, Sulaymaniyah Surgical Teaching Hospital, Zanko Street, Sulaymaniyah 46001, Iraq; Kscien Organization for Scientific Research (Middle East Office), Hamdi Street, Sulaymaniyah 46001, Iraq; Department of Urology, Sulaymaniyah Surgical Teaching Hospital, Zanko Street, Sulaymaniyah 46001, Iraq; College of Medicine, Department of Clinical Sciences, University of Sulaimani, Madam Mitterrand Street, Sulaymaniyah 46001, Iraq; Smart Health Tower (Raparin Branch), Karux Street, Ranya 46012, Sulaymaniyah, Iraq; Department of Urology, Smart Health Tower, Madam Mitterrand Street, Sulaymaniyah 46001, Iraq; College of Medicine, Department of Clinical Sciences, University of Sulaimani, Madam Mitterrand Street, Sulaymaniyah 46001, Iraq; Department of Radiology, Smart Health Tower, Madam Mitterrand Street, Sulaymaniyah 46001, Iraq; Department of Urology, Smart Health Tower, Madam Mitterrand Street, Sulaymaniyah 46001, Iraq; Department of Scientific Affairs, Smart Health Tower, Madam Mitterrand Street, Sulaymaniyah 46001, Iraq; Kscien Organization for Scientific Research (Middle East Office), Hamdi Street, Sulaymaniyah 46001, Iraq; Department of Scientific Affairs, Smart Health Tower, Madam Mitterrand Street, Sulaymaniyah 46001, Iraq; Kscien Organization for Scientific Research (Middle East Office), Hamdi Street, Sulaymaniyah 46001, Iraq; College of Medicine, Department of Clinical Sciences, University of Sulaimani, Madam Mitterrand Street, Sulaymaniyah 46001, Iraq; Department of Scientific Affairs, Smart Health Tower, Madam Mitterrand Street, Sulaymaniyah 46001, Iraq

**Keywords:** retained foreign body, kidney, delayed complication, secondary renal stones, percutaneous nephrolithotomy, penetrating trauma

## Abstract

Retained renal foreign bodies (FBs) following blast or firearm injury are rare and usually present early; delayed presentation is a challenging diagnostic scenario. The present report describes a case of retained renal shrapnel presenting with obstructive complications 18 years after the initial injury. Imaging revealed a 24 mm calculus encrusting a retained metallic FB at the left renal hilum, with severe hydronephrosis and reduced differential renal function (27.4%). Following ureteroscopic stenting, percutaneous nephrolithotomy was performed. The surrounding calculus was fragmented, but the metallic core resisted both pneumatic and laser lithotripsy and required extraction via a dorsal lumbotomy. A focused review of seven prior cases revealed an injury-to-presentation latency of 5 months to 17 years; the present 18-year interval is among the longest reported. Retained renal FBs may remain silent for years and closely mimic ordinary calculi; a thorough trauma history is essential to avoid misdiagnosis and guide operative planning.

## Introduction

Firearm and blast-related injuries remain a major source of morbidity, with the genitourinary system involved in ~10% of gunshot injuries [1]. The kidney is considered the most commonly injured genitourinary organ, involved in 1%–20% of all trauma cases [2]. The initial tissue response involves hematoma formation and acute inflammatory changes [3]. When projectiles lodge within the renal parenchyma without immediate communication with the collecting system, they may become encapsulated by fibrous tissue and remain inert for extended periods [4]. However, retained metallic fragments are not always biologically inactive and can instead elicit a chronic inflammatory response characterized by granulation tissue formation, scarring, and eventual fibrous encapsulation [5]. Foreign bodies (FB) within the urinary system are uncommon, and renal retention in particular remains especially rare. The etiology of retained renal FBs is usually iatrogenic, with traumatic implantation reported less frequently [6]. The present report describes a rare case of retained renal shrapnel presenting with delayed obstructive complications 18 years after the initial trauma. All sources were carefully evaluated for reliability and relevance, and the manuscript was prepared according to Case Report and Literature Review (CaReL) guidelines [7, 8].

## Case report

### Patient information

A 59-year-old male military personnel member presented with left loin pain, with no associated frank haematuria or dysuria. His past medical history was significant for hypertension and diabetes mellitus. His surgical history was notable for a retained left renal FB sustained from a shrapnel injury 18 years earlier, following which he underwent urgent laparotomy for abdominal trauma and a subsequent unsuccessful attempt at removal via an open left loin incision.

### Physical examination

On abdominal examination, a midline laparotomy scar and a left subcostal flank incision were noted; the abdomen was soft and non-tender, with no palpable masses.

### Diagnostic approach

**Figure 1:**
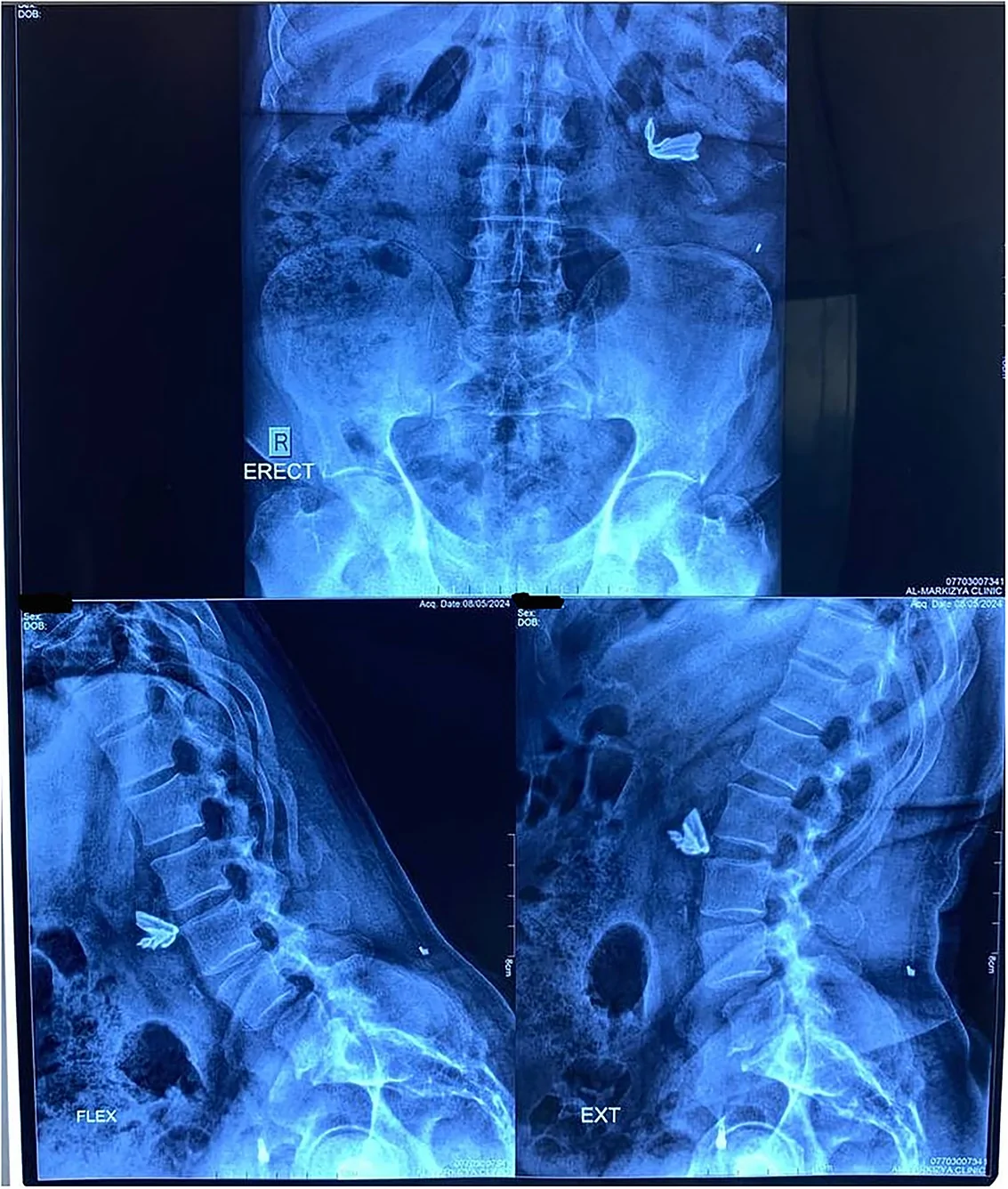
Kidneys, ureters, and bladder (KUB) radiograph, lateral view, demonstrating a radiopaque object on the left side of the abdomen at the level of the kidney.

**Figure 2:**
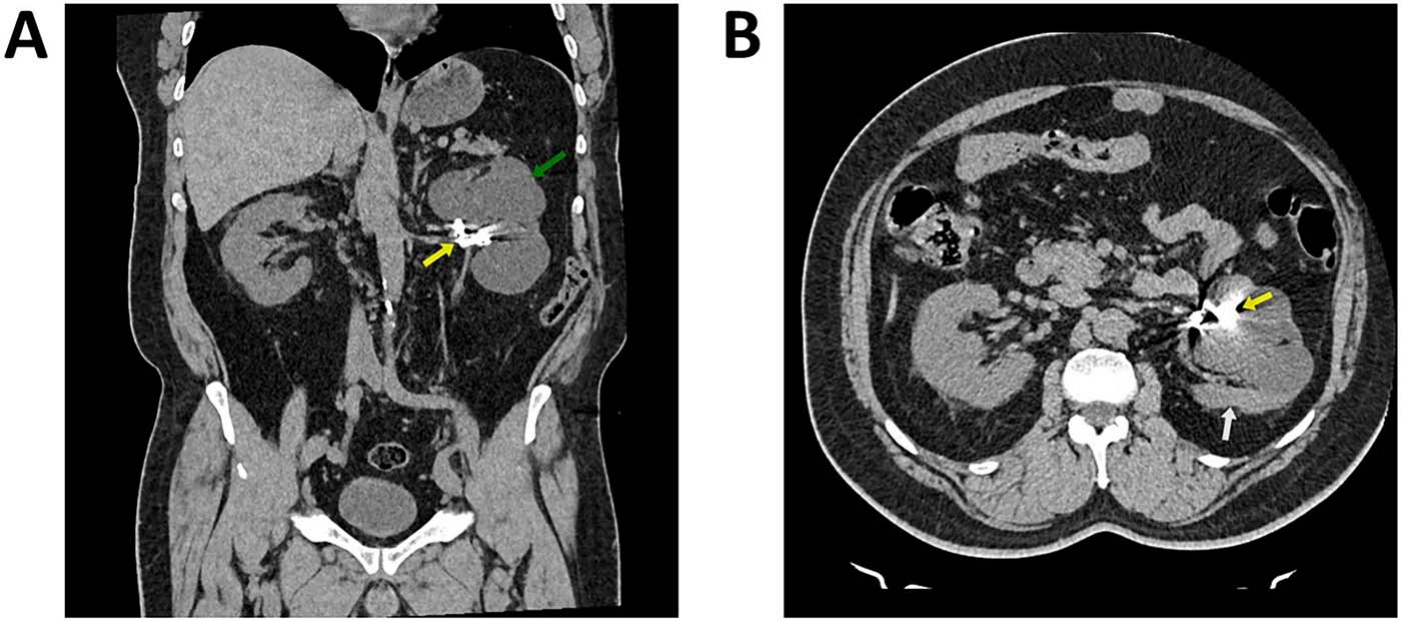
Native abdominal CT. A. Coronal section. B. Axial section. It shows severe hydronephrosis of the left kidney (green arrow), a small part of parenchyma is remaining posteriorly (white arrow), other parts show marked renal parenchymal loss, the cause is a metallic foreign body impacted in the UPJ and renal pelvis.

Initial laboratory investigations revealed a normal complete blood count and a serum creatinine of 1.1 mg/dL. General urine examination showed no pyuria but revealed 55 red blood cells per high-power field. A kidneys, ureters, and bladder radiograph revealed a radiopaque object on the left side of the abdomen (Fig. 1). Non-contrast computed tomography (CT) demonstrated a 24 mm stone with severe pelvicalyceal dilatation and cortical wasting, and metallic FBs at the left renal hilum obscuring the ureteropelvic junction (UPJ) and limiting visualization of the stone (Fig. 2). Dimercaptosuccinic acid scintigraphy showed a differential renal function of 27.4% for the left kidney.

### Therapeutic intervention

**Figure 3:**
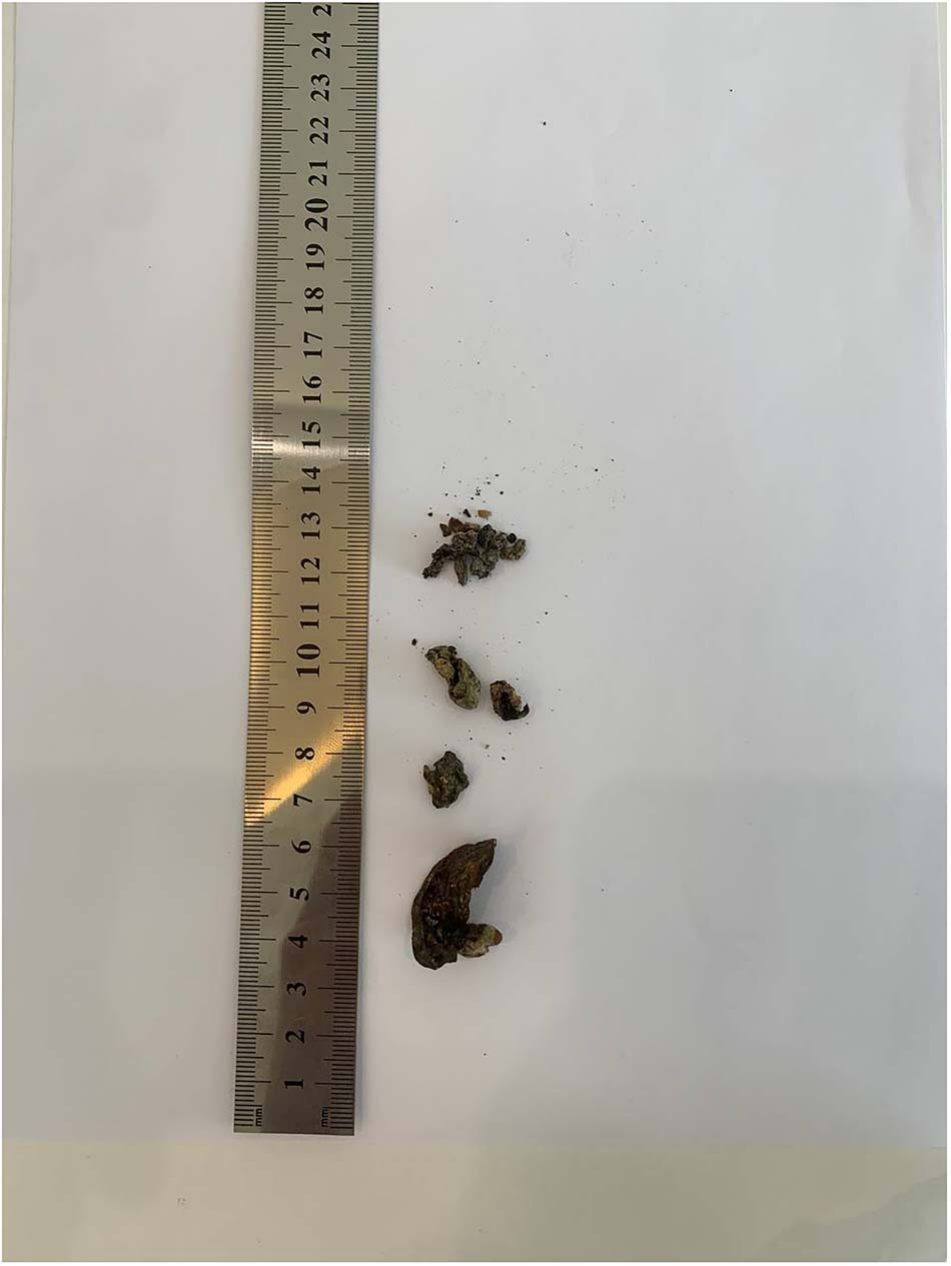
The removed metallic piece and the stone fragments.

Given two prior open abdominal procedures, percutaneous nephrolithotomy (PCNL) was selected to avoid previously operated planes. Following ureteroscopic placement of a ureteric stent, a single lower pole puncture was performed via a markedly dilated calyx, with tract dilation up to 30 Fr and placement of an Amplatz sheath. A large pelvic stone was fragmented; however, this revealed a metallic core consistent with retained shrapnel lodged within the renal pelvis. The fragment could not be disintegrated using either pneumatic or laser lithotripsy. Accordingly, a dorsal lumbotomy was performed along the Amplatz sheath tract to access the fragment, which was successfully extracted (Fig. 3).

### Postoperative course and follow-up

**Figure 4:**
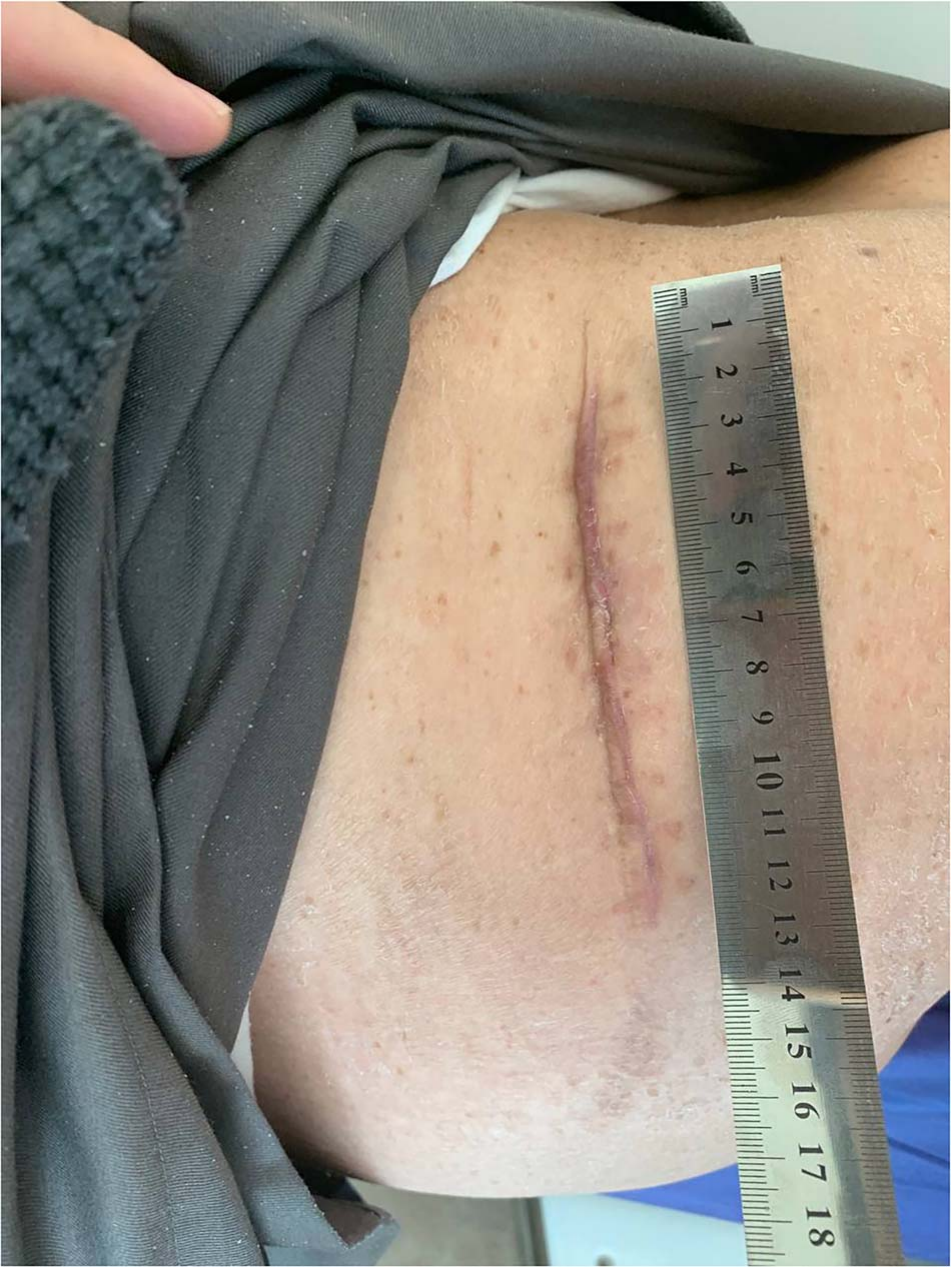
The site of the flank incision 1 month post-operatively.

The postoperative period was uneventful, and the patient was discharged in stable condition. One month after the procedure, the patient was clinically stable, the incision was well-healed, and the JJ stent was removed (Fig. 4).

## Discussion

Retained genitourinary FBs from warfare or firearm injury are rarely reported. Regardless of etiology, they may remain clinically silent for months to years before manifesting as urinary stones, obstruction, or lower urinary tract symptoms [9]. Diagnosis can be challenging, as FBs are easily misidentified as a conventional TeXcalculus or go undetected until intervention [5]. Late complications of retained renal projectiles are rare, with most symptomatic cases presenting early in the post-traumatic period [10].

A review of the literature revealed seven cases of traumatic retained FBs managed surgically after a prolonged interval from injury, excluding iatrogenic and ingested cases. All patients were male (aged 21–53 years). Bullets were the most common FB (five cases, 71.4%), with single cases each of a retained artillery shell fragment and a plastic detonating cap. Latency ranged from 5 months to 17 years (median 10 years). Stone formation occurred in 5 cases (71.4%), and PCNL was the primary approach in 6 (85.7%), with the remaining case managed by laparoscopic ureterotomy (Table 1) [4, 5, 10–14].

**Table 1:** Reported cases of retained renal traumatic foreign bodies managed with delayed surgery

Author, Year	Age/Sex	Clinical presentation	Latency	FB Type	Imaging	Anatomical location	Baseline renal Status	Management	Stone formation	Follow-up	Ref
Haseeb & Ali, 2024	28/M	Dull, aching right flank pain associated with nausea and vomiting.	10 years	Bullet	**X-ray (KUB)**: Showed multiple radiopaque shadows. **CT**: Differentiated renal stones (1310 HU) from a higher-density bullet (3100 HU).	Right renal pelvis.	Solitary right kidney (left absent)	PCNL with pneumatic lithotripsy; bullet grasped and removed	Yes (stone over bullet)	Asymptomatic at 1 month follow up	[11]
Kaestner *et al.*, 2021	23/M	Right flank pain, generalized abdominal pain, pyrexia, jaundice, and mental confusion.	5 months	Bullet	**Initial CECT:** bullet retained in right kidney (mid-zone). **Repeat CT** at 5 months: bullet migrated to right distal ureter with hydroureter and hydronephrosis **Ultrasound**: Noted echogenic kidneys. **X-ray**: after double-J stent placement.	Initially right kidney mid-zone; migrated to right distal ureter.	Bilateral grade III renal injuries	Double-J stent insertion followed by laparoscopic ureterotomy one month later	No (migration only)	NR	[12]
Naeem *et al.*, 2009	33/M	Left renal colic.	17 years	Artillery shell fragment	**CT-KUB**: Identified a 1 cm stone with high-density "sparkling artefacts" typical of metal.	Left mid-pole kidney.	Normal left kidney	PCNL via middle calyx; metal fragment and stone removed en toto without fragmentation	Yes (partial encrustation)	NR	[4]
Wang *et al.*, 2023	34/M	Asymptomatic and hemodynamically stable; requested removal of the bullet	6 months	Bullet	**Plain CT**: Increased density; **3D Reconstruction**: Precise localization; **Ultrasound**: Strong echo with acoustic shadow in the renal parenchyma	Right renal parenchyma	Normal	Ultrasound-guided percutaneous nephroscopy using a **1470 laser** to dissociate the bullet from surrounding tissue for extraction	None identified; bullet was embedded in granulation and scar tissue	5 years follow up: relatively satisfying RFT	[5]
Bissas *et al.*, 2005	21/M	Intermittent left-flank pain.	12 years	Plastic detonating cap	**KUB/IVU/CT**: Showed a stone-like opacity in the renal pelvis and lead shots in the parenchyma. **CT**: Differentiated the lead shots by a "star-like pattern".	Left renal pelvis.	Normal left kidney	PCNL; partial fragmentation followed by intact removal with rigid forceps	Yes (calcium epitaxis)	NR	[10]
Jhaveri & D'Angelo, 2018	53/M	Left flank pain	10 years	9 mm bullet	**CT**: Showed hydronephrosis and a 15 mm calcification. **Pyelography**: Identified a filling defect consistent with a calculus. **Fluoroscopy**: Visualized the fragment and a ureteral stent.	Left ureteropelvic junction (UPJ).	Solitary left kidney (right atrophic)	Ureteral stenting followed by PCNL with ultrasonic + holmium laser; bullet extracted via Amplatz sheath	Yes (stone encasing bullet)	NR	[13]
Tegegne *et al.*, 2025	29/M	Bilateral flank pain (worse on the right).	3 years	Bullet fragment	**Ultrasound**: Showed bilateral nephrolithiasis and moderate hydronephrosis of the right kidney. **CT**: Identified a metallic fragment by its "star-like pattern" and a secondary 1.46 cm stone.	Right upper pole infundibulum.	Normal right kidney (left also had stones)	Ultrasound-guided PCNL; pneumatic lithotripsy; forceps removal	Yes (secondary nephrolithiasis)	1 month: pain-free, site healed; hydronephrosis resolved	[14]

Abbreviations: CT, computed tomography; KUB, kidneys, ureters, bladder; IVU, intravenous urography; UPJ, ureteropelvic junction; HU, Hounsfield units; CECT, contrast-enhanced computed tomography; RFT, renal function tests; NR, not reported; PCNL, percutaneous nephrolithotomy; FB, foreign body; mm, millimeter; cm, centimeter.

A thorough history was crucial here: the shrapnel injury 18 years earlier and two prior laparotomies guided preoperative diagnosis and surgical planning. Similarly, Jhaveri and D'Angelo identified a bullet encased in stone only after correlating CT findings with the patient’s remote gunshot history, informing the selection of an appropriate endoscopic approach [13]. Computed tomography is the gold standard for renal FB diagnosis, and the characteristic star-like artifact of metallic objects has been used to identify a retained fragment within the collecting system [14].

Although the FB was correctly identified in the current case through history and imaging, renal FBs can be mistaken for ordinary stones. This challenge was illustrated by Bissas et al., in whose case the FB went unrecognized preoperatively, leading the patient to undergo two unsuccessful shockwave lithotripsy sessions before the definitive diagnosis was established postoperatively [10].

Management of the encrusted fragment is equally demanding. Haseeb and Ali used pneumatic lithotripsy to fragment the calculus around a retained bullet and extracted it through a 28 Fr Amplatz sheath [11]. In the present case, a larger 30 Fr sheath was used, but the shrapnel could not be extracted once the surrounding stone was fragmented and required a dorsal lumbotomy for retrieval. Naeem et al. instead avoided lithotripsy entirely, removing an artillery shell fragment en toto [4].

This report is limited by one-month follow-up, precluding assessment of long-term renal recovery, and by the absence of blood lead measurement, which limits evaluation of systemic toxicity.

## Conclusion

Retained renal FBs following blast or firearm injury may remain silent for years before presenting with delayed complications, and can closely mimic conventional urinary calculi. A thorough clinical history is essential to avoid misidentification and to guide operative planning.

## Data Availability

The data supporting the findings of this case report are included within the article. Additional anonymized clinical data are available from the corresponding author upon reasonable request.
